# How Modification of Accessible Lysines to Phenylalanine Modulates the Structural and Functional Properties of Horseradish Peroxidase: A Simulation Study

**DOI:** 10.1371/journal.pone.0109062

**Published:** 2014-10-14

**Authors:** Leila Navapour, Navid Mogharrab, Mehriar Amininasab

**Affiliations:** 1 Biophysics and Computational Biology Laboratory, Department of Biology, College of Sciences, Shiraz University, Shiraz, Iran; 2 Institute of Biotechnology, Shiraz University, Shiraz, Iran; 3 Department of Cell and Molecular Biology, School of Biology, College of Science, University of Tehran, Tehran, Iran; Instituto de Tecnologica Química e Biológica, UNL, Portugal

## Abstract

Horseradish Peroxidase (HRP) is one of the most studied peroxidases and a great number of chemical modifications and genetic manipulations have been carried out on its surface accessible residues to improve its stability and catalytic efficiency necessary for biotechnological applications. Most of the stabilized derivatives of HRP reported to date have involved chemical or genetic modifications of three surface-exposed lysines (K174, K232 and K241). In this computational study, we altered these lysines to phenylalanine residues to model those chemical modifications or genetic manipulations in which these positively charged lysines are converted to aromatic hydrophobic residues. Simulation results implied that upon these substitutions, the protein structure becomes less flexible. Stability gains are likely to be achieved due to the increased number of stable hydrogen bonds, improved heme-protein interactions and more integrated proximal Ca^2+^ binding pocket. We also found a new persistent hydrogen bond between the protein moiety (F174) and the heme prosthetic group as well as two stitching hydrogen bonds between the connecting loops GH and F′F″ in mutated HRP. However, detailed analysis of functionally related structural properties and dynamical features suggests reduced reactivity of the enzyme toward its substrates. Molecular dynamics simulations showed that substitutions narrow the bottle neck entry of peroxide substrate access channel and reduce the surface accessibility of the distal histidine (H42) and heme prosthetic group to the peroxide and aromatic substrates, respectively. Results also demonstrated that the area and volume of the aromatic-substrate binding pocket are significantly decreased upon modifications. Moreover, the hydrophobic patch functioning as a binding site or trap for reducing aromatic substrates is shrunk in mutated enzyme. Together, the results of this simulation study could provide possible structural clues to explain those experimental observations in which the protein stability achieved concurrent with a decrease in enzyme activity, upon manipulation of charge/hydrophobicity balance at the protein surface.

## Introduction

Horseradish peroxidase (HRP) is a Classical Secretory plant peroxidase isolated from horseradish (*Armoracia rusticana*) roots which catalyzes the oxidation of a wide variety of substrates, using hydrogen peroxide or organic peroxides [1], [2]. HRP C, the most studied heme peroxidase, consists of a single polypeptide chain of 308 amino acid residues, including a heme prosthetic group, two calcium ions, four disulfide bridges, and eight carbohydrate chains [3], [4]. The first crystal structure of glycan-free recombinant HRP has been reported in 1997 [5]. Since then, the three-dimensional (3D) structure of catalytic intermediates and several substrate complexes of HRP have also been reported and subjected to molecular dynamics (MD) simulations [6]–[12]. Some major advances in understanding the structural and functional aspects of HRP C have been achieved through these simulations, including the role of key residues in substrate binding, reactivity and stability [10]–[12].

HRP is one of the most extensively studied peroxidases mainly due to its diagnostic, biosensing, bioremediation, and other biotechnological applications [13]–[17]. Hence, many efforts have been made to improve the stability and activity of HRP through genetic and chemical modifications. Most efforts have been focused on targeting surface accessible residues; among them solvent-exposed lysines have been frequently mutated and successfully improved the enzyme stability [18]–[32]. Several reports indicate that modification of lysine residues in a number of other enzymes such as α-amylase, aminotransferase, trypsin, papain and stem bromelain also increases the enzyme stability [33]–[37]. On the other hand, modification of residues such as histidine, arginine, tyrosine, glutamic and aspartic acid leads to decreased or similar-to-native HRP stability [38], [39].

HRP contains six lysine residues, among them K174, K232, and K241 are located on the protein surface [3]. It has been reported that thermostability of HRP increases by chemical modification of these three accessible lysines, but the stabilizing effect is lost after complete modification of all six lysine residues [18].

Most of the stabilized derivatives of HRP reported to date have involved chemical or genetic manipulations that neutralize or reverse the positive charges on lysine residues. Accordingly, these modifications can be classified into three distinct groups: The first and second are those which alter lysines to neutralized aromatic or aliphatic hydrophobic residues. The third group, are charge-reversing modifications. In this study, we modeled a case from the first group by mutating the most accessible lysine residues to phenylalanine. In genetic manipulations, it is possible to target a specific residue, but there is no exact control over the chemical modifications and the accessibility of residues determines the rate and location of modifications. Moreover, it has been shown that increasing the number of modified lysine residues up to three increases the stability gain in HRP [18]. To mimic the case globally, all the three accessible lysine residues were modified simultaneously.

In earlier studies, we reported covalent attachment of an electron relay (anthraquinone 2-carboxylic acid) to the surface-exposed lysine residues of HRP. Experimental results showed that the modification alters all three accessible charged lysines and improves electron transfer properties, catalytic efficiency, and stability of the enzyme [29], [30]. MD simulations clarified some structural changes relating to stability and activity enhancement, including decreased flexibility of the protein backbone and redistribution of hydrophobic patches at the protein surface as well as peroxide and aromatic binding sites [30]. Here, we try to study a different case in which accessible charged lysines (K174, K232, and K241) are substituted by aromatic hydrophobic phenylalanine residues. We use MD simulation to investigate the structural and dynamical consequences of these substitutions.

## Materials and Methods

All Molecular dynamics simulations were performed using the GROMACS simulation package version 4.0.7 [40] and GROMOS96 force field [41]. The starting atomic coordinate of native HRP (n-HRP) was obtained from Protein Data Bank (PDB: 1ATJ) [5]. To generate the initial structure of the modified protein, p-HRP, the lysine residues 174, 232, and 241 of native HRP were modified to Phenylalanine residues. Each protein was centered in a cubic box and immersed in SPC water molecules so that the shortest distance between the protein and the box boundaries was 1.3 nm and periodic boundary conditions were applied. To achieve a neutral simulation box, the net charge of the protein was neutralized by replacing water molecules with necessary Cl^−^ or Na^+^ ions. Each solvated and neutralized system was energy-minimized using the steepest descent algorithm until the maximum force was smaller than 500 kJ/mol.nm. After energy minimization, two separate position-restrained MD simulations were sequentially carried out to adjust temperature and velocities and to equilibrate the solvent and ions around the protein. First, to adjust the system temperature, an NVT MD simulation was performed for 200 ps at 300 K by imposing thermal energy in a constant volume condition using the velocity rescale algorithm (modified Berendsen thermostat) with τ_T_ = 0.1 ps [42], [43]. After arrival at the correct temperature, the resulting atom velocities and coordinates were used to start an NPT MD simulation at 300 K and 1 bar for 200 ps by the Parrinello-Rahman algorithm with τ_P_ = 2.0 ps during which density of the system was stabilized at around 1000 kg/m^3^ [44]. Finally, the production MD period of 100,000 ps at constant pressure (1 bar) and temperature (300 K) without position restraints was performed on native and p-HRP. Bond lengths were constrained using LINCS algorithm [45]. In Gromos96 force field, the Lennard-Jones and short-range electrostatic interactions are optimized with a cut-off radius of 1.4 nm. However, in order to reduce the computation time, we used a shorter cut-off of 1.0 nm based on the tested insensitivity of HRP simulation down to this value. The particle mesh Ewald algorithm was used for the long range electrostatic interactions [46]. The neighbor list was updated every 5 steps. Each component of the system was coupled separately to a thermal bath, and isotropic pressure coupling was used to keep the pressure at the desired value. A time step of 2 fs was used for the integration of equation of motion.

In order to evaluate the quality or sufficiency of conformational sampling of simulations, the production MD period was divided into four 25 ns parts and principal component analysis was performed on each sub-trajectory. Eigenvectors and eigenvalues were obtained from the diagonalization of the covariance matrices of the C_α_ atoms, and the principal components were generated by projecting the trajectories on the respective eigenvectors [47]. The cosine content of the principal components was calculated to estimate whether the conformational fluctuations are connected with the potential (when the cosine content is close to 0) or with random diffusion (when the cosine content is close to 1) [48].

## Results

### Global Dynamics

**Figure 1:**
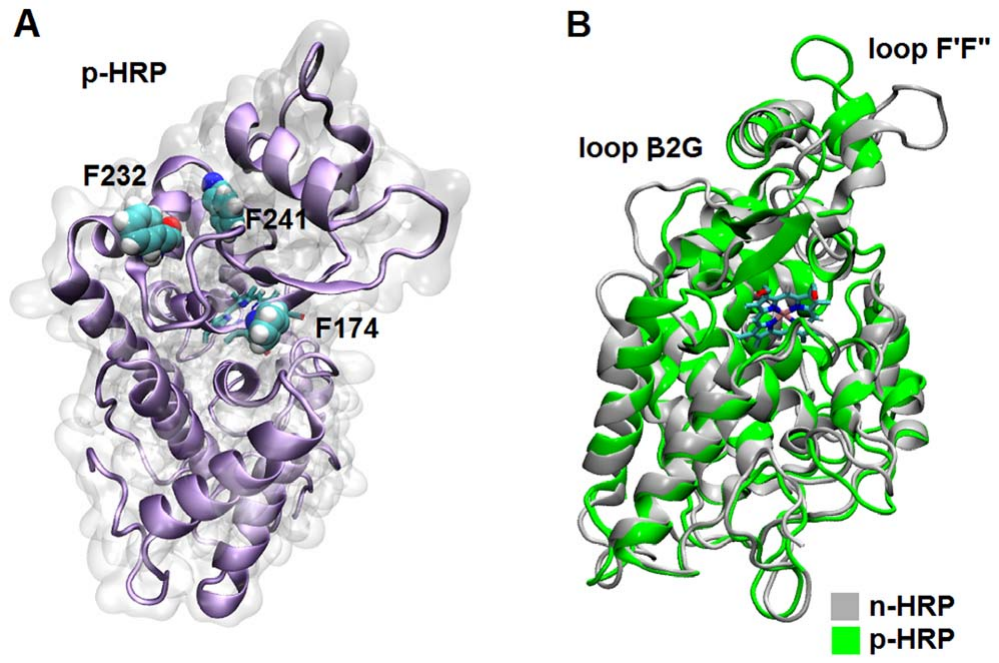
Comparative modeling of HRP. (A) 3D representation of the MD average structure of p-HRP: the molecular surface is rendered in transparent light gray to make the ribbon model visible. (B) Superimposition of the average structures: the structures of n-HRP (gray) and p-HRP (green) obtained by averaging over the analyzed time frames. Major differences were observed in the connecting loop β1F′, helix F′, connecting loop F′F″, strand β2 and the connecting loop β2G.

**Figure 2:**
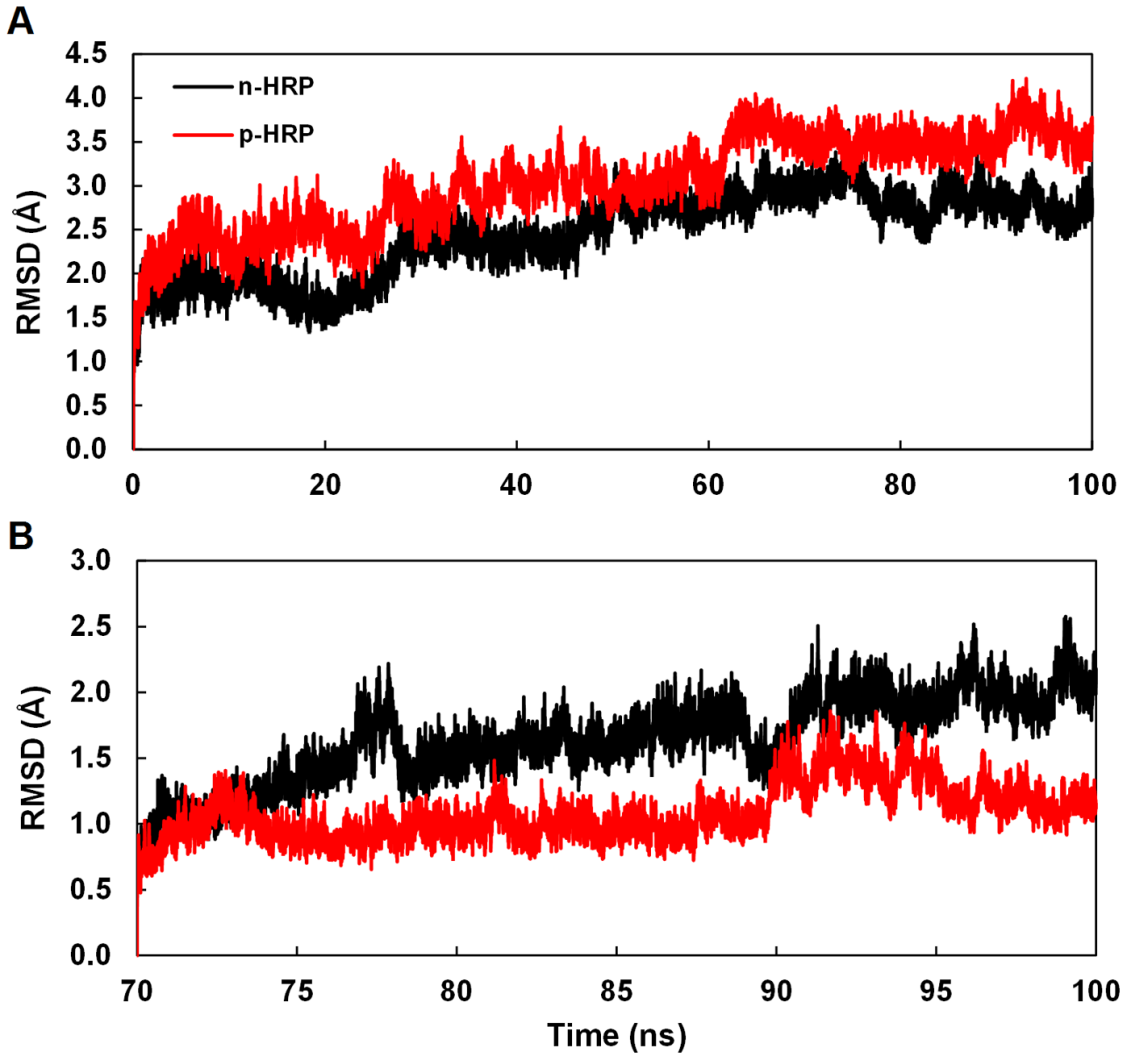
Time dependence of the backbone RMSD. (A) Backbone RMSD with respect to the starting structure during the entire course of 100 ns MD simulations. (B) Backbone RMSD relative to the structure of 70th ns (i.e. the starting structures of the analyzed time frames) during the last 30 ns MD simulations. Those of the n-HRP are reported in black and those of the p-HRP are shown in red.

Using the known x-ray crystallographic structure of HRP C (PDB: 1ATJ), two 3D molecular models of HRP C, n-HRP and p-HRP, were constructed differing in the residues 174, 232, and 241 (Fig. 1). Keeping the amino acid pattern of the original X-ray structure, the control model (n-HRP) was built with cationic lysines at these positions. In the next model (p-HRP) charged lysine residues were substituted by hydrophobic aromatic phenylalanine residues. The overall stability and structural relaxation of the enzymes were monitored by computing time evolution of the root mean square deviation (RMSD) of the backbone atoms along the simulations. The RMSD of n-HRP and p-HRP with respect to their starting structure were 2.46±0.47 and 3.03±0.52 Å, respectively. This indicates that the structure of mutated protein undergoes more conformational changes along the simulation. Comparison of the RMSD per residue profiles of the MD trajectories revealed that these changes are concentrated in the N terminal part of the protein structure (including residues 1–12), C terminal part of the loop D′E (residues 140–144) and the helix E (residues 145–153) (data not shown). For n-HRP model, the backbone RMSD as a function of time reaches a relative plateau after about 50 nanoseconds (ns) of simulation, but for p-HRP such a plateau is achieved after about 70 ns (Fig. 2A). Hence, to be statistically comparable, our analyses were focused on those trajectories obtained from the last 30 ns of simulations for both the native and mutated proteins. In order to check whether the sampling of conformational space is sufficient, the production MD period was divided into four 25 ns parts and the cosine content of the first 4 principal components was calculated for each sub-trajectory. For both n-HRP and p-HRP models, the best results of cosine content were obtained for the last quarter part of the 100 ns simulations. The values of the first 4 principal components for this time window were calculated to be 0.146, 0.459, 0.036, 0.110 for n-HRP and 0.197, 0.517, 0.010, 0.001 for p-HRP.The stability of the fluctuation of the potential energy was also examined by calculating the ratio between the variance and average of potential energy. This ratio for n-HRP and p-HRP was respectively about 0.00099 and 0.00092, thus showing that energy was conserved during the simulations and providing additional evidences indicating that the simulations and models were stabilized.

**Figure 3:**
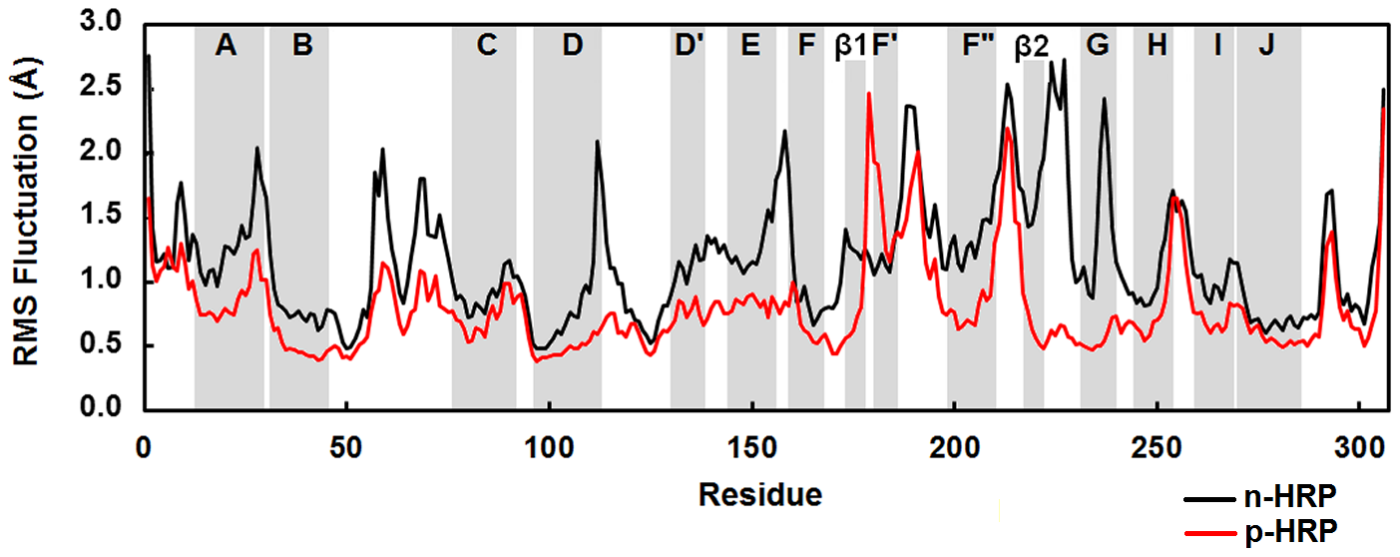
Structural flexibility of simulated models. Backbone RMSF per residue reported for n-HRP (black) and p-HRP (red) along the analyzed time frames. The gray bands indicate the helix and sheet regions of HRP according to the crystallographic structure.

In the last 30 ns of simulations, the backbone RMSD of n-HRP and p-HRP structures relative to their own 70th ns structures (i.e. the starting structures of the analyzed time frames) was calculated to be 1.64±0.33 and 1.08±0.21 Å, respectively (Fig. 2B). In agreement, the root mean square fluctuation (RMSF) values for this time window also show a clear decrease upon substitutions (Table 1), implying reduced flexibility of the protein backbone as a result of these mutations. To provide a more detailed description of the mobility of protein residues, the backbone RMSF per residue for the native and mutated HRP is shown in Fig. 3. According to the crystallographic structure (PDB: 1ATJ), HRP contains thirteen α-helices and two short antiparallel β-strands. It is obvious that the mobility of regular secondary structures, except for the helix F′ (including residues 181–183), is mainly reduced after substitutions. Reduced mobility is particularly significant in helices F″ and G, as well as the strands β1 and β2. Apart from regular structures, mobility of the connecting loop β2G is also greatly diminished. Furthermore, the mobility of protein backbone around the heme prosthetic group is declined from 1.10 Å in n-HRP to 0.69 Å in p-HRP. Taken together, it can be concluded that the overall backbone structure has become less flexible upon these mutations. Therefore, decreased mobility of the heme prosthetic group is to be expected (Table 1).

**Table 1:** Summary of the enzyme structural properties obtained from the analysis of the last 30 ns MD simulations.

	n-HRP	p-HRP
**RMSF (Å)**		
Protein backbone	1.17	0.80
Protein backbone around Heme	1.10	0.69
Heme	1.14	0.69
**Surface area (nm^2^)**		
Hydrophobic area	82.34±1.80	76.23±1.80
Hydrophilic area	84.92±1.88	78.34±1.51
**Volume (nm^3^)**	54.13±1.47	53.26±1.39
**Radius of gyration (Å)**	20.33±0.15	20.09±0.09

**Figure 4:**
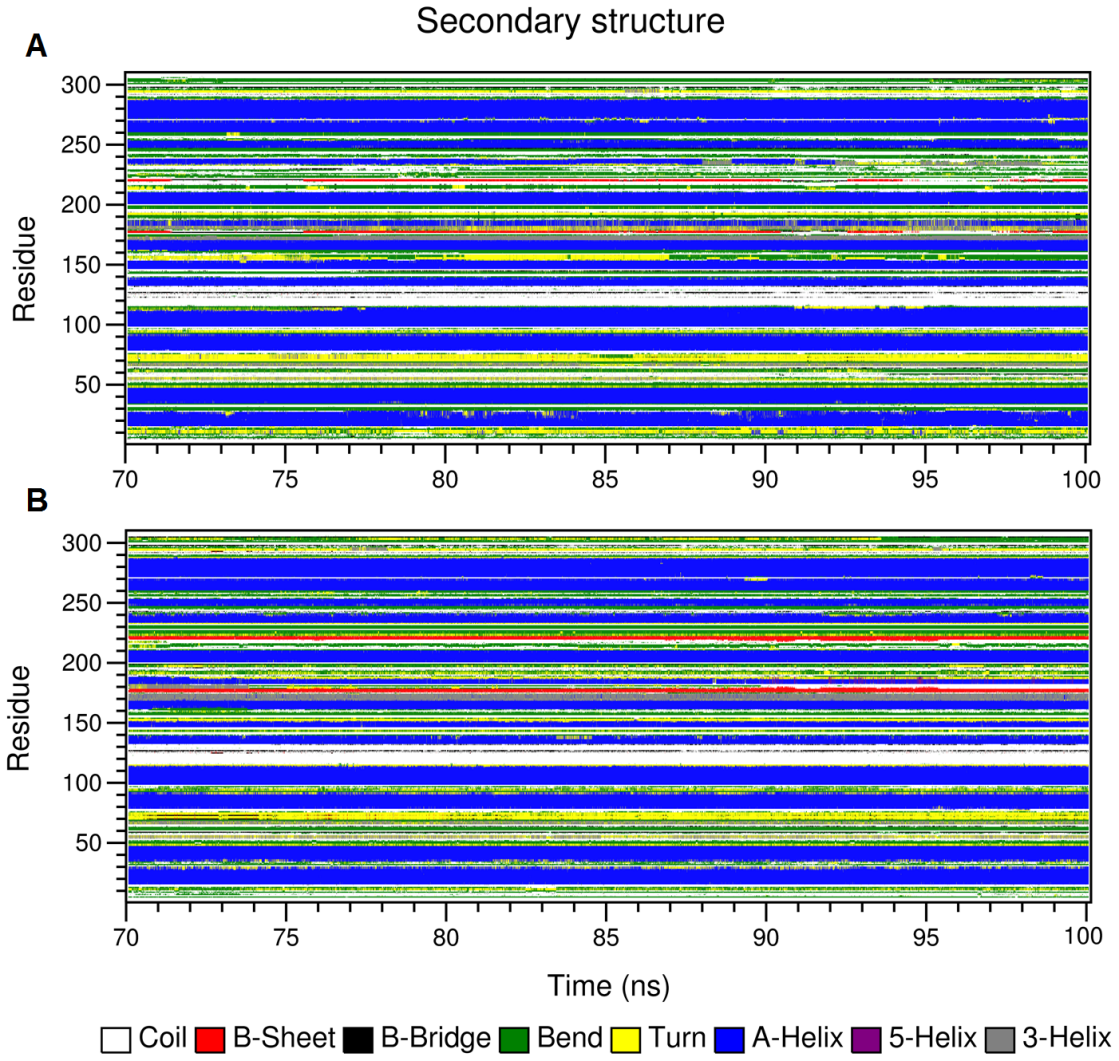
Secondary structure analysis of the native and modified HRP models. Time-dependent secondary structure fluctuations of n-HRP (A) and p-HRP (B) models calculated using the DSSP program. The occurrence of secondary structure elements is indicated by using a color code.

Additional information on the structural flexibility is offered by the analysis of time-dependent secondary structure fluctuations. The secondary structure content of the models was calculated as a function of time using the DSSP program [49] (Fig. 4 and Table 2). Analysis of Fig. 4 reveals that in both models, α-helices exist persistently throughout the simulations, although the values in Table 2 indicate a small increase in the α-helical content of p-HRP which is mainly due to the stability of two short helices F′ (residues 181–184) and G (residues 231–239). By comparing the occurrence of these helices along the simulations (Fig. 4), it becomes clear that both of them, particularly helix G, are more persistent in p-HRP. It is noteworthy to remind that the substituted residue 232 resides in this helix. In addition, the helix patterns assigned by DSSP show subtle differences between the two models. The end of the helix A is extended by three residues (Arg-27, Ser-28, and Asp-29) in p-HRP compared with n-HRP. A similar trend was also observed in the helix D during the first 5 ns of analyzed time frames.

**Table 2:** Occurrence of secondary structure elements calculated from the last 30 ns of n-HRP and p-HRP simulations.

	n-HRP	p-HRP
**Coil,%**	24.84	24.27
**β-Sheet,%**	0.84	2.08
**β-Bridge,%**	1.77	1.89
**Bend,%**	19.59	17.75
**Turn,%**	8.64	9.24
**α-Helix,%**	41.03	41.55
**5-Helix,%**	0.001	0.12
**3-Helix,%**	3.28	3.09

In order to gain a better insight into the effects of these substitutions on HRP structure, local changes around the substitution sites were also investigated. As mentioned earlier, HRP contains a β-sheet comprised of two short antiparallel β-strands, β1 and β2, which flank the helices F′ and G, respectively. K174 is located in strand β1, at the proximal side of the heme cavity. DSSP analysis of the MD trajectories shows that while in p-HRP, the β-sheet structure is maintained almost throughout the entire analyzed time frames, it is absent in 36.25% of the n-HRP trajectories and replaced by β-bridge approximately in one third of the simulation time. This is in accordance with the observed increase in existence percentage of those hydrogen bonds connecting backbone of the residues involved in β-sheet formation in p-HRP model (Table 3A). It seems that improved hydrogen bonding in this region accounts for lower mobility of the strands β1 and β2 in modified HRP compared with the native one. In addition to the changes mentioned above, K174F improves hydrogen bonding between protein moiety and the heme prosthetic group. Backbone amide of Phe-174 forms a strong hydrogen bond (occupancy 98.93%) with heme O2D propionate oxygen, whereas in n-HRP this hydrogen bond is almost absent. Furthermore, this substitution strengthens the hydrogen bonds of Ser-35 and Arg-31 with propionate oxygens of the heme prosthetic group. Together, these findings suggest that Phe at position 174 may have a local stabilizing effect on p-HRP structure.

**Table 3:** Existence probability of hydrogen bonds involved in the formation of β-sheet and helix G during the analyzed time frames.

	Existence percentage, %	
	n-HRP	p-HRP
**A) β-Sheet forming hydrogen bonds**		
Asn175N Asn175H Val219O	57.8	99.3
Cys177N Cys177H Ala217O	67.9	88.5
Val219N Val219H Asn175O	74.1	91.3
**B) Helix G forming hydrogen bonds**		
Val235N Val235H Asn231O	17.4	65.6
Asn236N Asn236H Lys/Phe232O	64	99.1
Leu237N Leu237H Tyr233O	47.4	93.7
Glu238N Glu238H Tyr234O	―	90.7
Glu239N Glu239H Val235O	―	45.1

Residues 232 and 241 are located in the helix G and the connecting loop GH, respectively. Helix G is longer by three residues in p-HRP than in n-HRP (Fig. 4). On the other hand, the data presented in Table 3B suggests that this helix is more stable in p-HRP due to the formation of more and stronger hydrogen bonds. Replacing Lys-232 with Phe also improves the hydrogen bonding strength between Phe-232 and Asp-230, Thr-171 and Asp-230, Asp-230 and Ile-228, Thr-225 and Asp-222 which are involved in proximal Ca^2+^ coordination (Table 4). This substitution also introduces a new hydrogen bond between Asn-236 and Asp-222 that are bound to Phe-232 and proximal calcium ion, respectively. Such changes would enhance the stability of proximal Ca^2+^ binding pocket. Moreover, the orientation of the connecting loop β2G between helix G and β-sheet has changed after modification. In p-HRP, formation of a new hydrogen bond between the side chain nitrogen of Asn-236 in helix G and the backbone oxygen of Asp-222 in loop β2G pulls the loop β2G towards the helix G. Also in p-HRP, Gln-240 and Gly-242 (from the connecting loop GH) which flank the substitution site 241 form two persistent hydrogen bonds with Asn-198 and Thr-196 (from the connecting loop F′F″), respectively. These new hydrogen bonds stitch end of loop F′F″ to the beginning of loop GH and enhance the integrity of this part of p-HRP structure.

**Table 4:** Existence probability of hydrogen bonds between some residues involved in proximal Ca^2+^ coordination during the analyzed time frames.

		Existence percentage, %	
Donor	Acceptor	n-HRP	p-HRP
Thr171OG1	Asp230O	42.9	98.2
Thr225OG1	Asp222OD2	41.4	99.4
ASP230N	Ile228O	14.8	61
Lys/Phe232N	Asp230OD1	29.8	57
ASN236ND2	Asp222O	0	64.8

Comparison of the structures also reveals that the connecting loop β1F′, helix F′, connecting loop F′F″ and helix F″ (residues 177–208) exhibit considerable conformational changes (Fig. 1B). The relative orientation of this part of p-HRP model, especially the connecting loop F′F″, is quite different from that of the crystal structure, while no significant deviation in this part of structure was observed for n-HRP. The connecting loop F′F″ rotates from F′ side in n-HRP to the F″ side in p-HRP. Although through this rotation some hydrogen bonds are broken or weakened, they have been compensated by new ones in p-HRP. For example, while the hydrogen bond between the backbone nitrogen of Asp-194 and the backbone oxygen of Arg-183 is absent in p-HRP, a compensating hydrogen bond between Arg-183 and Thr-196 has been formed in the modified model.

To assess the effect of mutation of three positively charged lysine residues on the exposure pattern of surface charge/hydrophobicity, the water accessibility of hydrophobic and hydrophilic surface areas in each model was measured and averaged over the analyzed time frames. Surprisingly, both the hydrophobic and hydrophilic exposed areas are harmoniously declined in p-HRP (Table 1), so as the ratios between the areas remain almost identical after substitution (0.9696 in n-HRP and 0.9731 in p-HRP). Consistent with the total surface area, Table 1 shows that the average values for radius of gyration and total protein volume are also decreased upon substitution. Comparing the average distance between two structural calcium ions shows a decrease from 31.42±2.03 Å in n-HRP to 28.24±0.30 Å in p-HRP. This finding is particularly important because the substrate binding cleft in HRP is located between the two calcium ions and any closing of this cleft could profoundly affect the heme availability and consequently the enzyme activity.

**Figure 5:**
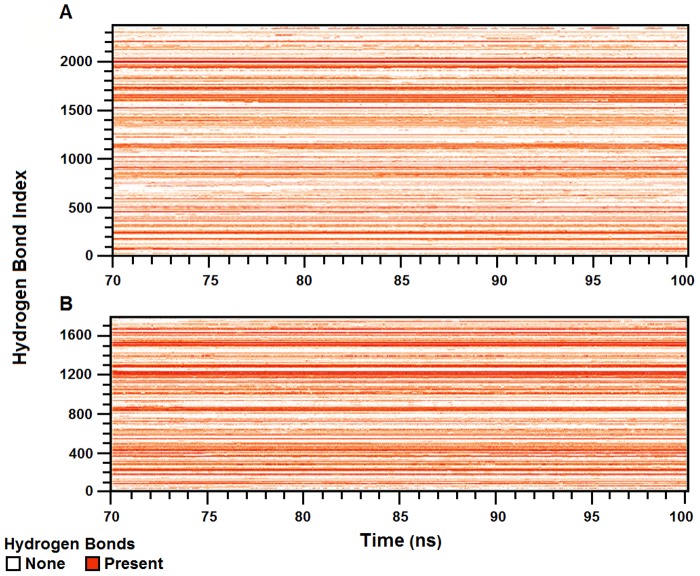
Hydrogen-bond existence map for n-HRP (A) and p-HRP (B) along the analyzed time frames. Red lines show the presence of a hydrogen bond at that specific time and white breaks imply the absence of that hydrogen bond.

Fig. 5 shows the existence map of hydrogen bonds for n-HRP and p-HRP models over the corresponding time frames of simulations. Analysis of the internal protein hydrogen bonds evinces that the number of stable hydrogen bonds is increased in p-HRP. For example, the total number of intramolecular hydrogen bonds with existence percentage greater than 80% has increased from 103 in n-HRP to 115 in p-HRP (Table 5). In agreement, the percentage occupancy of hydrogen bonds forming β-sheet and helix G in p-HRP is significantly higher than that of n-HRP (Table 3). Together, these findings suggest that consolidation of intramolecular hydrogen bonding network actively contributes to the stability enhancement of p-HRP.

**Table 5:** Hydrogen bond existence probability during the analyzed time frames.

Existence percentage	n-HRP	p-HRP
**90%≤**	78	84
**80%≤**	103	115
**70%≤**	130	136
**60%≤**	154	156
**50%≤**	183	180
**40%≤**	213	218
**30%≤**	243	262
**20%≤**	297	304
**10%≤**	390	394
**5%≤**	508	482
**1%≤**	874	788

### Peroxide-binding site

**Figure 6:**
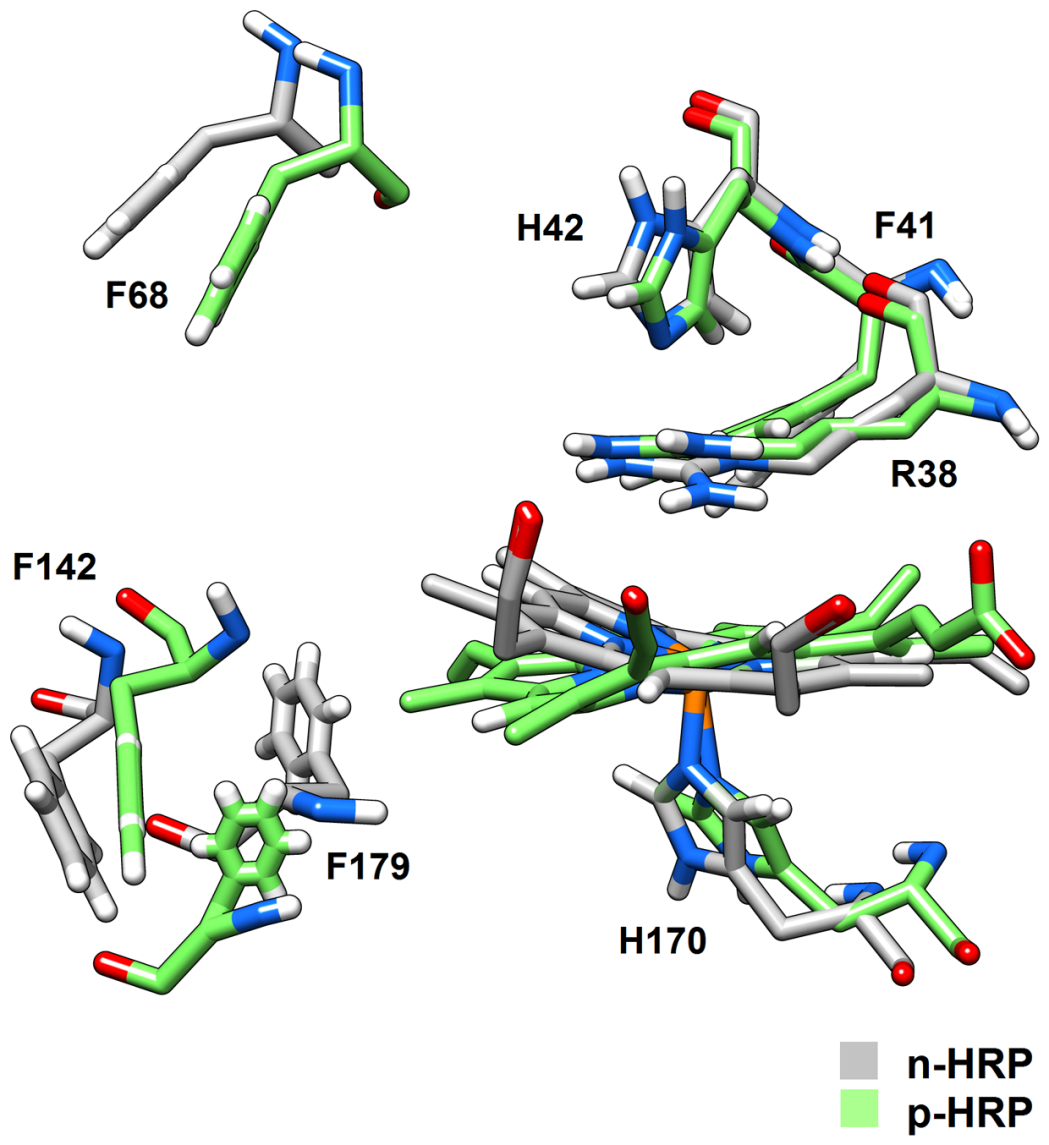
Heme pocket alignment. Structural features of the heme crevice reported for n-HRP (gray) and p-HRP (green) according to their MD average structures. Phe residues 68, 142, and 179 guard the entrance to the exposed heme edge and control substrate access to the active site in HRP. Especially compare the distance between Phe-68 and Phe-142 in MD structures.

HRP utilizes hydrogen peroxide (or organic peroxides) to oxidize a broad range of organic and inorganic substrates. The main overall reaction catalyzed by HRP can be summarized as H_2_O_2_ + 2AH → 2H_2_O + 2A*, where AH represents a reducing substrate and A* is a free radical product. Hydrogen peroxide oxidizes the native enzyme to an intermediate that called Compound I, which then can oxidize the reducing substrate [2]. To react with the heme prosthetic group, hydrogen peroxide permeates the protein at a fluctuating entry point located between Phe-68 and Phe-142. Conformational fluctuations of these Phe residues determine the accessibility of hydrogen peroxide to the interior [10]. In comparing the two simulated models, the average distance between the backbones of Phe-68 and Phe-142 decreases from 12.08±0.95 Å in n-HRP to 10.64±0.54 Å in p-HRP (Fig. 6). Accordingly, the average distance between their side chains decreases from 12.19±1.87 Å to 10.23±1.21 Å. Examination of the RMS fluctuations of these residues also reveals significant decreases in p-HRP. The RMSF of Phe-68 and Phe-142 has declined from 1.80 and 1.22 Å in n-HRP to 1.08 and 0.84 Å in p-HRP, respectively. These values indicate that the bottleneck entry has become tighter and less flexible upon substitution. These effects are expected to be less sensible for small peroxides and significant for bulky ones.

In the proposed mechanism for the reaction of H_2_O_2_ with the active site of HRP two essential features are acid-base catalysis by the distal histidine (His-42) and charge stabilization of a precursor enzyme-substrate complex by the conserved distal arginine (Arg-38) [50], [51]. The histidine is thought to facilitate formation of the initial iron-peroxide complex by deprotonating the peroxide and subsequently promoting cleavage of the oxygen-oxygen bond by protonating the distal oxygen [50]. Comparing the models shows that the surface accessibility of this histidine in p-HRP (8.07±4.88 Å^2^) is significantly less than n-HRP (23.95±9.62 Å^2^). This change could lead to decreased reactivity of the modified enzyme to peroxide substrates.

### Aromatic substrate-binding site

According to the catalytic cycle of HRP, the first step is the oxidization of the native enzyme to a short-lived intermediate, called compound I, by hydrogen peroxide or organic peroxides. In two subsequent single electron-transfer steps, the oxidized intermediate is initially reduced to a second intermediate, called compound II, and finally to the resting state of the enzyme through reactions with two reducing substrate molecules [2], [13]. Reduction of compound II to the native enzyme is the rate limiting step in the catalytic cycle of HRP [52], [53]. To react with compound II, the aromatic reducing substrate should diffuse from the bulk solution to the aromatic-binding pocket (i.e. the heme cavity). Substrate oxidation by HRP C occurs at the exposed heme edge, a region comprising the heme methyl C18 and heme meso C20 protons [2]. Spectroscopic and crystallographic studies have revealed a detailed picture of the site where aromatic substrates bind and react with the protein. A ring of three peripheral Phe residues 68, 142, and 179 guards the entrance to the exposed heme edge (Fig. 6). Amino acid residues Phe-68, Gly-69, Leu-138, Pro-139, Ala-140, Pro-141, Phe-142, and Phe-179 are flanking the substrate access channel and together with the heme C20- and C18-methyl groups form the aromatic-binding pocket of HRP [5]. Although in some cases hydrogen bonding between the reducing substrate and the active site residues of the distal heme pocket contributes to the stability of the substrate-HRP complex, most HRP substrates do not possess the potential to make such interactions and will therefore depend more on the hydrophobic interactions which characterize the peripheral region of the substrate channel of HRP [54]. Accordingly, besides the size of reducing substrates, two determinant factors could affect the reaction rate; volume of the active site cavity which controls the penetration of reducing substrates into the active site and the extent of the hydrophobic patch functioning as a binding site or trap for reducing aromatic substrates which is related to the enzyme affinity for the aromatic substrates. Our results show that both factors are affected upon the substitutions. As mentioned before, comparison of the RMSD per residue profiles of the MD trajectories revealed that in p-HRP, the C terminal part of the loop D′E (residues 140–144) and the helix E (residues 145–153) undergo more conformational changes along the simulation (data not shown). Phe-142 of the loop D′E is located at the opening of the heme crevice and together with other residues of this loop (Leu-138, Pro-139, Ala-140 and Pro-141) covers one side of the inner wall of substrate access channel. Upon substitutions, conformational changes which occur in the loop D′E push these residues and the subsequent helix E toward the inside of the heme cavity, making the substrate access channel and its opening narrower in p-HRP. At the same time, the strand β1 (residues 174–176), which is located at the proximal side of the heme cavity and holds one of the substituted residues, also moves toward the inside of the heme crevice, so as to tighten it even more. Together, these movements make the active site cavity smaller in p-HRP. To evaluate this effect, average structure of each model was submitted to CASTp server (Computer Atlas of Surface Topology of protein) and the volume of the active site cavity was measured [55]. CASTp calculations showed that the area and volume of the active site cavity (i.e. the aromatic substrate binding pocket) are decreased from 675.2 Å^2^ and 960.2 Å^3^ in n-HRP to 441.4 Å^2^ and 591.3 Å^3^ in p-HRP, respectively. These changes are expected to hinder the access of bulky aromatic substrates to the heme active site and so affect the rate limiting step in the catalytic cycle of HRP. In agreement, the values in Table 6 show that hydrophobic residues forming the substrate-binding pocket in HRP are generally less exposed in p-HRP, implying that the hydrophobic patch functioning as a binding site or trap for reducing aromatic substrates is shrunk in the mutated enzyme. Values in Table 6 also indicate that the accessibility of the exposed heme edge has been significantly reduced after mutations. Summing up the above simulation results, the conclusion is reached that such conformational changes at the aromatic substrate-binding site of HRP may contribute in reducing the affinity and reactivity of the enzyme for aromatic substrates. Again, the effects are expected to be less sensible for small substrates and significant for bulky ones.

**Table 6:** Solvent accessible surface area (Å^2^) of functionally important residues and groups averaged over the analyzed time frames.

	n-HRP		p-HRP		
Residue	Average	SD	Average	SD	Change
**His-42**	23.95	9.62	8.07	4.88	−15.88
**Phe-68**	127.95	40.52	143.68	25.05	15.73
**Gly-69**	25.87	12.81	12.32	6.00	−13.55
**Leu-138**	28.64	9.32	15.39	6.21	−13.25
**Pro-139**	37.74	13.59	10.42	5.90	−27.32
**Ala-140**	51.00	11.08	24.82	7.25	−26.18
**Pro-141**	60.76	14.07	31.20	14.28	−29.56
**Phe-142**	80.55	18.44	119.03	16.62	38.48
**Phe-179**	59.79	14.92	82.74	31.33	22.95
**Heme**	151.59	34.08	80.62	17.09	−70.96

## Discussion

Directed mutagenesis and chemical modification have been frequently used to characterize or stabilize HRP. HRP directed mutagenesis has been applied primarily to identify those amino acids which are critical for catalysis, structure maintenance, or stability of the enzyme. A detailed description of the introduced point mutations and their effects can be found in reviews [2], [15], [56], [57]. However, genetic engineering tools have also been used to improve HRP stability. One of most extensive studies has been carried out by Ryan and Ó'Fágáin, who described 22 HRP mutants to evaluate their stability against hydrogen peroxide. Most mutants displayed little or no alteration in H_2_O_2_ stability, while three mutants (K232N, K241F and T110V) exhibited significantly increased H_2_O_2_ tolerance, among them, the K241F single point mutation enhanced hydrogen peroxide tolerance about 12-fold [26]. In another study, they analyzed the effects of 13 single and 3 double point mutations on the stability of HRP. Three single mutants (K232N, K232F, K241N) demonstrated increased stability against heat (up to 2-fold) and solvents (up to 4-fold) [27]. These studies indicate that single substitution of either K232 or K241 by Phe residues is beneficial for the stability of HRP structure, although it cannot be inferred that simultaneous mutation of these two is additive or synergistic. There are also some reports indicating that modification of solvent-exposed lysines 174, 232 and 241 improves HRP stability [20], [58], while exhaustive modification of all six lysines of HRP leads to decreased stability [18]. Accordingly, in this study, we replaced these surface exposed lysines of HRP by phenylalanine, in order to evaluate their simultaneous effects on HRP structure. In agreement to the aforementioned experiments, our simulations provide some structural clues implying that simultaneous substitution of these three lysines by phenylalanine residues can improve the enzyme stability.

Genetic engineering tools are not the only way to modify HRP properties. Actually, chemical modifications have been used more frequently to stabilize HRP. Clearly, chemical modification of solvent-exposed lysines 174, 232 and 241 has produced the best results. Chemical modification of these lysines, ranging from the use of a cross-linker through attachment of polyethylene glycol to simple acetylation, has succeeded in stabilizing HRP to varying degrees [18]–[25], [28]–[32]. A comprehensive review has been published covering the structure and physicochemical properties of the modifiers and their effects on HRP stability [59], although more recent studies are also available [28], [31], [32]. Perhaps, one of the first and most extensive studies is reported by Ugarova et al., who studied thermostability of HRP after modification of its lysyl amino groups with a variety of modifiers including anhydrides of monocarboxylic and dicarboxylic acids as well as picryl sulfonic acid. Some of these compounds neutralized the positive charge on the lysine, whereas others reversed it. Authors concluded that modification of three out of six lysine residues produces the major effect on macromolecular conformation and thermostability of HRP [18].

In the case of this study, three accessible charged lysine residues were replaced by hydrophobic phenylalanine residues. So, one can expect the enzyme surface to be more hydrophobic after substitution, but our simulation results disagree with this speculation. Both the hydrophobic and hydrophilic exposed areas are harmoniously declined in p-HRP, so as the ratio between the areas remains almost identical after substitution. Consistent with the total surface area, the average values of radius of gyration and the total volume of protein also decreased upon substitution (Table 1). Although the magnitude of these changes is not statistically significant, they show a similar trend of decrease along with increasing the compactness of protein structure.

Further analysis of the trajectories seeking the local effects of substitutions led us to several clues in the intramolecular hydrogen bonding network indicating stabilization of p-HRP; (1) Analysis of the internal protein hydrogen bonds evinces that the number of stable hydrogen bonds has increased in p-HRP. For example, p-HRP benefits from 12 more intramolecular hydrogen bonds with existence percentage greater than 80% as compared to n-HRP. (2) Hydrogen bonds forming the β-sheet (i.e. between the strands β1 and β2) and helix G in p-HRP are more persistent than those of n-HRP. (3) We also found a persistent new hydrogen bond between the protein moiety (Phe-174) and the heme prosthetic group (O2D propionate oxygen), as well as improvements in the probability of some pre-existing hydrogen bonds in p-HRP. This additional hydrogen bond could strengthen the heme-protein interaction. It is important because in plant and fungal peroxidases, the binding of the heme prosthetic group to the protein moiety greatly depends on non-covalent interactions. (4) The hydrogen bonding strength between some residues involved in proximal Ca^2+^ coordination shows considerable improvements upon substitutions (Table 4). Such changes could enhance the stability of proximal Ca^2+^ binding pocket which is assumed to be essential for the structural and functional integrity of the enzyme [27], [60]. (5) Formation of two persistent stitching hydrogen bonds between the connecting loops GH and F′F″ could enhance the integrity of this part of p-HRP structure. Although the contribution of these changes to the protein stability may not be great individually, the additive contributions of their small favorable enthalpic changes can produce an overall considerable enthalpic gain in stabilization of modified HRP.

In addition to the hydrogen bonding network, the results revealed subtle differences between the secondary structure content of the models. DSSP analysis of the MD trajectories showed that the helices F′ and G, as well as the β-sheet structure are more persistent in p-HRP. The structural stability of β-sheet in the modified model is in accordance with higher propensity of Phe for β-sheet conformation as compared to lysine [61], [62]. The end of the helix A is also extended by three residues in p-HRP. A similar trend was also observed in the helix D during the first 5 ns of analyzed time frames.

These substitutions also decrease the RMSD and RMSF values. Such observation has also been reported in experimentally stabilized derivatives of HRP. Ugarova et al. observed that in successfully stabilized derivatives, the intensity of the Soret band in CD spectra of HRP decreases [18]. Accordingly, they inferred that thermostability of the modified enzymes increases due to the decreased conformational mobility of the protein backbone around the heme. Similar changes in CD spectra of HRP after modification with maleic anhydride, citraconic anhydride and anthraquinone 2-carboxylic acid has also been reported [24], [30]. Our simulation results provide additional support for this inference, but interestingly indicate that this phenomenon is not merely limited to the heme pocket and the overall protein structure experiences a similar reduction in flexibility upon substitutions.

Mutagenesis and chemical modification of amino acids can also influence catalytic properties. To our knowledge, surface-exposed lysines of HRP were always modified or substituted with the aim of improving stability and in most reports, the activity of the stabilized forms of HRP has been described to be similar-to-wild type or with minor changes (see [59] and the references within), sometimes without making references to detailed kinetic parameters. Surprisingly, in some cases, precise evaluation of the reported data led us to a confusing conclusion. Actually, it seems that in modified HRPs, activity has attracted less attention than stability. This may arise from the fact that HRP has a very high catalytic turnover and some loss in this activity does not significantly affect its practical usefulness. For example, Ugarova et al. studied the effect of 12 charge-neutralizing and charge-reversing lysine modifiers on thermostability of HRP. Comparison of the temperature-activity profiles indicated that at any temperature between 20–55°C, the modified HRPs exhibit lower activity than the native enzyme [18]. Considering their experimental measurements, the following conclusions can be inferred: (1) According to the reported temperature-activity profiles, at about 50°C, which is the temperature of HRP maximum activity, modified HRPs show about 20% less activity in comparison to the native enzyme. (2) These profiles also indicate that thermostabilized HRPs require more heat (up to 15°C) to achieve their maximum activity. (3) Their CD spectra imply that the conformational mobility has decreased and so they inferred that the protein moiety around the heme has become more rigid. Taken together, the conclusion is reached that upon stabilization, the enzyme becomes more rigid, loses some of its activity and needs more heat to acquire the flexibility required for similar to wild-type enzyme activity level. In agreement to these experimental observations, our simulation results showed that upon charge-neutralizing substitutions, the mobility of the protein structure as well as the volume of the reducing substrate binding cavity decreases and the corresponding access channel becomes narrower. Accordingly, lower affinity of the enzyme for reducing substrate is expected. Experimental measurements of the apparent Michaelis constant (i.e. the K_m_ value) for both K232F and K241F single mutants are also in line with this conclusion, although the apparent *k_cat_* measurements indicate some increases [27]. Since the number of substitutions in these experimental studies and our simulations are not the same, some differences between their outcomes are not unexpected. In support of this conclusion, it is noteworthy to mention that Ugarova et al. has shown that the number of lysine modification produces a major effect on the macromolecular conformation [18].

Among the charge-neutralizing lysine modifications, it has been reported that modification of HRP with glucosamine hydrochloride [63] and anthraquinone 2-carboxylic acid [29], [30] generates not only more stable but also catalytically more active enzymes. In the case of chemical modifications, it is difficult to compare the results of these studies with our Lys-to-Phe substitutions, because the structure and physicochemical properties of these modifiers differ significantly from that of Phe side chain. Glucosamine is a highly hydrophilic carbohydrate having several hydroxyl groups [63]. Attachment of anthraquinone 2-carboxylic acid (a tricyclic aromatic electron relay) to the side chain of lysine residue forms a bulky side chain which differs greatly from that of Phe in size and hydrophobicity [29], [30]. Such differences in structure and physicochemical characteristics could result in substantial variations in outcomes.

In conclusion, the results of this simulation study indicate that hydrophobization of the surface exposed lysines of HRP does not necessarily lead to an unfavorable change in the charge/hydrophobicity balance at the protein surface and may produce stabilized derivatives with acceptable changes in enzyme activity. We have no evidence to think that this phenomenon is merely limited to the surface exposed lysines. Therefore, it would be a good idea to check whether substitution of phenylalanine for the other surface-exposed charged residues (apart from lysines) is beneficial for HRP stability. More generally, the results could also provide possible structural clues for understanding how manipulation of charge/hydrophobicity balance at the protein surface could be translated into structural and functional changes within a protein.
